# Notes on Egypt as a Health-Resort

**Published:** 1895-12

**Authors:** P. Watson Williams

**Affiliations:** Assistant-Physician to the Bristol Royal Infirmary


### NOTES ON EGYPT AS A HEALTH-RESORT.

BY

P. Watson Williams, M.D. Lond.,

*Assistant-Physician to the Bristol Royal Infirmary.*

The Northern African seaboard has long been recognised as a region which presents many and great attractions as a winter resort for sufferers from various affections of the chest, and which is, at the same time, easily accessible to Europeans. Certain regions, more especially Algiers, have long successfully competed with the Riviera for the patronage of French and English patients who are compelled to exchange the cold and damp of their own less favoured winter for a more genial climate.

Egypt is a land of all-engrossing interest, both in its historic remains and in the varied phases of modern life that are ever before the traveller's eye; and as it is so rapidly becoming one of the most popular winter resorts, I have endeavoured, in the narrow limits of the present article, to indicate the various routes by which it may be reached, the character of the climate, cost of living, and the various affections for which it is most suitable, together with other practical points which may aid the practitioner in choosing a health-resort suited to the condition of health and the means of his patients.

For those who greatly dislike the sea voyage, Alexandria is the best port of entry, as it is only six days journey from London (by Paris, thence to Marseilles, fourteen hours, Messagerie steamboat to Alexandria, three days); or, going overland to Brindisi, the P. and O. boat can be taken for Alexandria. After a night's rest, one proceeds to Cairo by the morning express, a three hours run. Another route has recently been provided by the P. and O. Company by boat from Venice to Port Said, or by the Orient line from Naples to Port Said, five days. The long sea route from London or Plymouth to Port Said is often to be preferred for those who are good sailors—ten days at sea. One can sleep at Port Said if the boat arrives at night; otherwise it is better to go on to Ismailia, four hours by rail, where there is an excellent inn, now taken over as a succursal to Shepheard's Hotel (Cairo); or, if the patient is strong enough, the remainder of the journey by rail to Cairo may be accomplished in another four hours.

In Egypt everyone is, or should be, a "Cook's tourist," there is then no fear of being pestered by the clamorous crowd of native porters. The ubiquitous "Cook" boards the steamer, and one has only to hand the luggage checks to the agent, and mention the name of the hotel one is going to, without giving it another thought. If one cares to convert a peaceful journey into one long and incessant worry by officious natives who understand nothing but Arabic, unless it is a very big D——, one has only to try a journey without "Cook."[1]

Cairo has about 430,000 inhabitants, and on leaving the new and handsome station to be driven to one of the many large first-class hotels, we soon realise that, with all the brilliant pictures of Oriental life which surround us on every side, we have landed in a city where all the comfort and luxury of home life obtain. At almost all the first-class hotels in Cairo, Mena House, and at Luxor and Assouan, board and lodging come

[1] I have found that patients who have fixed a date for their passage, and are unable to go, are allowed to postpone it without further payment.

to about sixteen shillings a day, or less (fourteen shillings and sixpence) by the month, this including everything except wine, washing, and light. At Helouan Hotel the charge is 8/6 to 10/6 a day, while at the very comfortable *Pension* Antonio for those who are not strictly invalids the inclusive charge is only seven shillings a day.

I do not propose to discuss at any length the relative merits of various winter resorts, but in the briefest manner to direct attention to the peculiar conditions of Cairo and its neighbourhood, and the reasons for regarding Egypt as eminently suitable for English patients.

It is altogether beyond the scope of a short and practical article to portray the endless sources of interest and attraction of Cairo and its neighbourhood, but it is obvious that these constitute by no means unimportant features in an ideal health-resort, and the fact that there is always plenty to see and to think about, serves to distract the thoughts and overcome the depressing effects of an enforced removal from home. Moreover, a land which can show us "in the flesh" both Seti, and Rameses II. "who oppressed the children of Israel" and held intercourse with Moses; the beautiful and perfectly preserved gold and enamelled jewellery of the days of Joseph; and the Pyramids, which were of an older date to Abraham when he arrived on the scene than Westminster Abbey is to us, yet displaying at least a greater development of engineering skill than does our own more beautiful abbey, constantly brings home to us the fact that 7000 years ago Egyptians had reached a very high degree of intelligence and civilisation, and is a never-failing source of interest to every visitor.

It is only within the last eight years, however, that Egypt has developed as a health-resort for English invalids. The highly successful administration of the country under the British occupation has resulted in widespread reforms in sanitation, and in giving an English complexion to the hotel and private life of a large European population. Not only is the impress of British ideas of wholesome sanitation manifest in every direction in the large towns, but it extends to the villages around Cairo and up the Nile in a less degree. As a good illustration of this, I

may say that while living in a village in the desert it was astonishing to find that a small Oriental *pension* had been fitted up with the National flush sanitary appliances. On the other hand it must be confessed that even in some European quarters in the large towns there is much to be desired in various important sanitary departments, and there is too often good reason to regard water only as "the stuff we wash in."

*Climate*.—The climate of Egypt during the season from November to March is much the same as a moderately hot English summer, with perpetual sunshine during the day, and cool evenings; but with this difference, that after the subsidence of the Nile, the remarkable dryness of the atmosphere renders the air bracing and tonic, and prevents the sense of oppression that one so often experiences in the moist heat of an English summer.

The following table, compiled by Dr. F. W. Sandwith[1] from the records of the five years 1884—88, the monthly bulletins being printed by the Khedivial Observatory in the Abbassiyeh suburb of Cairo, shows at a glance the mean temperature in this region, the dryness of the atmosphere, and the almost complete absence of rain and stormy wind:—

| | Barometer. | Thermometer Fahr. | | | Humidity. | | Rain in inches. | Clouds 0—10. | Wind. | |
|---|---|---|---|---|---|---|---|---|---|---|
| | | Mean of maxima. | Mean of minima. | Mean of means. | Relative. | Absolute. | | | Direction. | Force in miles. |
| January ... ... | 29.98 | 61.4 | 46.6 | 53.6 | 69.7 | .28 | .19 | 4.1 | S.W. | 2.2 |
| February ... ... | 29.95 | 65.3 | 48.8 | 57 | 66.2 | .29 | .24 | 4.2 | N. | 1.4 |
| March ... ... | 29.91 | 73.2 | 53 | 62.8 | 56.2 | .29 | .03 | 3.4 | N. | 2.5 |
| April ... ... | 29.82 | 81.2 | 59.9 | 70.4 | 47.8 | .31 | .12 | 3.4 | N. | 2.6 |
| May ... ... | 29.84 | 86.8 | 63.4 | 75.2 | 48.4 | .38 | .22 | 2.3 | N. | 2.8 |
| June ... ... | 29.78 | 94.7 | 70.2 | 82.6 | 44 | .44 | .02 | 1 | N. | 3 |
| July ... ... | 29.7 | 93 | 72.2 | 83.8 | 49 | .5 | — | 1.2 | N. | 4.3 |
| August ... ... | 29.7 | 92.9 | 71.4 | 82.2 | 55.3 | .54 | — | 1.6 | N. | 4.1 |
| September ... | 29.83 | 87.5 | 68 | 77.8 | 62.1 | .54 | — | 1.8 | N. | 4.3 |
| October ... ... | 29.89 | 84 | 64.8 | 74.3 | 65.8 | .52 | — | 2.5 | N. | 3.2 |
| November ... | 29.96 | 74.2 | 56.3 | 64.4 | 67.5 | .39 | .21 | 3 | N. | 2.1 |
| December ... | 29.99 | 67.7 | 50.4 | 58.4 | 69.6 | .33 | .19 | 3.7 | N. | 2.2 |
| Average ... | 29.86 | 80.1 | 60.4 | 70.2 | 58.46 | .40 | 1.22 | 2.6 | N. | 2.9 |

But the remarkable even temperature of Cairo is perhaps more fully realised when we remember that "freezing point

[1] *Egypt as a Winter Resort*, 1889, p. 25.

seems never to be reached in Cairo, while the indoor temperature of rooms on the north side of the house, kept properly shut during the hot hours, need never, during the year, exceed 83° Fahr.; and the same rooms, during the depth of winter, if properly opened to receive the warm outer air during the day, need never have a temperature below 52° Fahr., without employing a fire. By living in sunny south rooms or by employing artificial heat, the invalid can be certain all night in the winter of a temperature of 65° Fahr. or more."[1]

We have seen that the annual rainfall of Cairo is a little over an inch. At Helouan and Gizeh it is even less; while at Luxor and Assouan a shower of rain has been known to occur only once in three or four years.

Peterson[2] compares statistics as to the annual rainfall in various resorts, although, as he remarks, this is not so important as the number of cloudy and rainy days at the different health stations during the season.

| | Annual Rainfall in inches. | Number of Rainy Days during 7 months. (Winter Season.) |
|---|---|---|
| Luxor ... ... | Practically *nil* | Usually *none* |
| Cairo ... ... | 1 | 12 |
| Hyères ... ... | 33.5 | 32 |
| Algiers ... ... | 32 | 71 (6 months) |
| Pau ... ... | 43 | 50 ,, |
| Denver ... ... | 11.41 | 39 |

During September and October the Nile rises and floods the low-lying ground on either side of its banks; but by the end of November the water has subsided, leaving immense tracts of moist, fertile land. At night the dew, which is always present in lower Egypt, is especially heavy during, and for a month after, "High Nile," so that invalids should avoid going out at night till the middle of December.

But the most remarkable feature of the Egyptian climate is the peculiar dryness of the atmosphere. At Cairo the mean relative humidity is 58.46; while even during the seven winter months it averages only 63.25, and in the daytime it is much lower, often reaching 10. At Gizeh and Helouan the humidity is considerably lower than at Cairo, the difference being still

[1] Sandwith, *op. cit.* [2] *Med. Rec.*, 1892, xlii. 205.

more remarkable at Luxor and Assouan. When compared with London, where the humidity averages about 92.0 in winter, the importance of these figures is beyond dispute. The following table[1] will illustrate the difference in mean annual humidity of various places in the world:—

| | |
|---|---|
| Luxor ... ... ... ... ... ... ... | 50.5 |
| Cairo ... ... ... ... ... ... ... | 58.4 |
| Greenwich ... ... ... ... ... ... | 87 |
| Algiers ... ... ... ... ... ... ... | 70.7 |
| New York ... ... ... ... ... ... | 73 |

*Prevailing Diseases, Hospitals, etc.* — Amongst the natives diarrhœa is common, and infant mortality from this cause is high. This is readily accounted for, as their ideas on sanitation are primitive to a degree. In native quarters water is collected and stored in skins, or in large earthen waterpots, which act as filters. The skins are handed down from father to son till they rot, and the filters are never cleaned; consequently their filthy and slimy condition reaches a state which can be more easily imagined than described. The water is taken direct from the Nile, and in one village I saw women washing clothes and men filling water skins and pots four feet from where the refuse of a donkey stable was running into the Nile, while the lapping margin of the river was continually washing away fæcal deposits on the bank. Fortunately the inhabitants seem to acquire a sort of immunity against the natural results from imbibing Nile water from such a source. In Cairo, as in Alexandria, the water is supplied by a company, which filters its water through large sand and gravel beds, and with subsequent filtration and boiling it becomes excellent.

Tubercular disease is by no means rare amongst the Arab and Coptic natives, while the Soudanese from the warmer and drier South are very subject to consumption, a large percentage of the deaths at the hospitals being due to pulmonary tuberculosis.

Malaria is not common, and, except that it not infrequently recurs in those who have suffered from previous attacks in India or elsewhere, Europeans rarely suffer; typhus fever is fairly common amongst the natives, but in the better classes

[1] Peterson, *loc. cit.*

it is rarely seen; the latter, on the other hand, suffer from enteric fever, which the natives rarely contract. Visitors are most subject to diarrhœa from careless exposure to night air with insufficient abdominal clothing, while pleurisy and pneumonia sometimes result from chills from similar want of due care.

An analysis of 690 cases admitted to one of the medical sections of the Kasr-el-Aini Hospital in 1894 (total admissions 4,090) throws some light on the relative frequency of certain diseases; viz., typhus, 19; relapsing fever, 1; enteric fever, 0; intermittent fever, 32; ankylostomiasis, 125; pellagra, 28; bilharziosis, 21; hepatic abscess, 10; hepatic cirrhosis, 4; aneurysm, 1; parenchymatous nephritis, 29; interstitial nephritis, 0; cerebral hemorrhage, 9; locomotor ataxia, 2; disseminated sclerosis, 7; general paralysis, 3; admitted drunk, 13; datura and hasheesh poisoning, 5; mercurial poisoning (suicidal), 1.[1]

Cairo is well supplied with hospitals, for in addition to the military hospital at the citadel, there is the above-mentioned Kasr-el-Aini Hospital, with about 400 beds, on which a large sum has been recently expended in partly rebuilding and in fitting up. Dr. Sandwith kindly showed me a large number of interesting cases under his care, and one was struck by the large number of ankylostomiasis cases in his wards. The clinical clerks are mostly native students at the medical school, and all the cases were recorded very carefully and fully. I saw Mr. Milton operating and demonstrating in the theatre to a large clinic. The arrangements of the operating theatre were excellent and left but little to be desired by the most captious critic. There is a good staff of English nurses under a most able matron.

There is also a large German hospital, and a small Medical Mission hospital with a large out-patient service in old Cairo which I visited. Private nurses are not difficult to obtain from a nursing home close to the Bab-el-Lûk station, where also a few private patients can be taken in.

The State Lunatic Asylum, with about 250 inmates, is conducted on modern methods. Dr. Peterson draws attention to the fact that insanity is phenomenally rare in Egypt, which with six million inhabitants has but this one asylum, whereas

[1] *Lancet*, 1895, ii. 560.

New York State with the same population has over fifteen thousand insane in its numerous asylums. For patients there are certain advantages in residing, during their stay in Lower Egypt, away from the noise and dust of Cairo, at either Gizeh or Helouan. There is only the English hotel, Mena House, at Gizeh, close to the Pyramid of Cheops. It is eight miles drive from Cairo in the desert, a coach running daily, while carriages can always be hired. Visitors will find everything that can be desired for their comfort at this large hotel. Dr. Bentley, the present physician to Mena House, resides in the hotel, which has a chapel and a resident chaplain, a large open swimming bath, arrangements for tennis, croquet, and golf, and there are rides and excursions from Mena House.

Helouan is fourteen miles by rail from Cairo, and is a small residential suburb, also in the desert, and contains two large and comfortable hotels; numerous *pensions* and houses may be rented at low rates. Dr. Fenyez resides in Helouan hotel, and a Scotch physician, Dr. Murison, lives in the village. The hot sulphurous springs at Helouan have long enjoyed a reputation for chronic rheumatic affections; they had a reputation in the days of the Pharaohs, thus Helouan is probably the most ancient spa known at the present day. The baths were built by Ismail Pasha, and are well constructed, resembling those at Aix-les-Bains. The springs are of three varieties: (*a*) the sulphur waters, of which there are four springs yielding 700 cubic metres daily, temp. 86° F., containing free sulphuretted hydrogen; (*b*) the iron waters; (*c*) alkaline waters. These waters are employed against secondary and tertiary syphilis, chronic skin diseases, hepatic congestion, &c.[1]

[1] The composition of the waters is approximately (Fenyez):

1. Sulphur Springs—

| | |
|---|---|
| Chloride of Calcium ... ... ... | 1.88 parts in 10,000. |
| " " Magnesium ... ... | 18.105 " " |
| " " Sodium ... ... ... | 366.892 " " |
| Free Sulphuretted Hydrogen ... | 0.731 (470 c.cm.) |
| " Carbonic Acid ... .. ... | 1.200 (610 c.cm.) |

2. Iron Springs—

| | |
|---|---|
| Chloride of Sodium ... ... | 372.67 |
| " " Magnesium ... | 106.02 |
| Sulphate of Magnesium ... | 23.5 |
| Bicarbonate of Lime ... ... | 59.42 |
| Bicarbonate of Iron ... ... | 0.58 |
| Free Carbonic Acid ... | .511 (260 c.cm.) |

3. Alkaline Waters—

| | |
|---|---|
| Chloride of Magnesium | 31.158 |
| " " Sodium ... | 40.17 |
| Sulphate of Magnesium | 10.79 |
| Bicarbonate of Lime ... | 12.56 |
| Free Carbonic Acid ... | 0.117 (60 c.cm.) |

Helouan being 185 feet above the Nile, surrounded by the desert and protected on the east by the Mokattam range of hills, has a remarkably dry and sterile atmosphere, and is rapidly becoming not only a favourite locality with invalids in Lower Egypt, but also a large residential suburb of Cairo.

At Luxor, first cataract, there are two comfortable hotels. The more fashionable Luxor hotel is close to a large sheet of water, and is probably less healthy in situation than the quieter and very comfortable Karnak hotel. Dr. Longmore and Dr. Canney are in practice here. Some patients go still further south to Assouan, about a thousand miles up the Nile from Cairo, where English medicine is well represented by Dr. R. W. Yalland. Luxor and Assouan are reached by boat, dahabiyeh, from Cairo. The dahabiyehs are very comfortably fitted up, and Thomas Cook and Sons make all arrangements, so that for a patient with ample means the journey to these far distant resorts is accomplished without difficulty or fatigue. Tourist steamers, which stop *en route* for visits to tombs and temples, and the more rapid post-boats are convenient and less expensive means of reaching Luxor and Assouan.

Alexandria itself cannot be called a health-resort, but patients returning from Cairo and Upper Egypt who are anxious to avoid the hot Khamseens, may spend the month of April at Ramleh, a suburb of Alexandria, till the weather is warm enough for them to go to the Riviera or to England. The Khamseens are hot winds or sand-storms. Khamseen is the Arabic for fifty, and the winds are thus named because they are liable to blow during fifty days shortly after Easter. The wind lasts from one to three days at a time, and there are about six to twenty Khamseen days altogether in the year. The wind is very dry and hot, and laden with very fine sand which penetrates into every room and covers everything. One would think that these sand-laden winds would be very trying to consumptives, but I was surprised to hear from an English clergyman, whose lungs were very extensively diseased, that he never felt better than when the Khamseen blew; "it seemed to clear his chest." According to Dr. Sandwith the general effects are, a little excitement and stimulation of the system, a more rapid succession of ideas, and increased action of some of the functions, followed by listlessness, headache, and languor.

This brief sketch of Egypt and its health-resorts will serve to show the nature of the climate and the kind of life that a patient or visitor may expect on his arrival. It will be noted that the Egyptian climate is far more to be depended upon than that of the Riviera, where cold and rain, and often frost, alternate with the days of sunshine, while the atmosphere is as dry as the higher Alps, without the disadvantage of cold and inaccessibility. Moreover, for many diseases the highly rarefied air of high altitudes, such as Davos and St. Moritz, is not altogether suitable.

The affections for which Egypt is best adapted may be briefly summarised as follows: Chest affections, particularly pulmonary tuberculosis, bronchitis, and asthma especially when complicated with bronchitis. Canney gives as pathological conditions in which the patient will derive great benefit: Phthisis: (1) non-erethic cases; (2) hemorrhagic cases; (3) non-acute cases of first or second stage, especially commencing deposit without very acute symptoms; (4) chronic quiescent phthisis, with or without cavities; (5) cases associated with bronchitis. Asthma, idiopathic and symptomatic; chronic bronchitis and emphysema; cases convalescent from pneumonia, pleuro-pneumonia, and pleurisy, where the structures have not returned to the normal state; chronic nasal and pharyngeal catarrh, and Eustachian deafness; cases of mental strain, breakdown, and irritability from overwork, especially such cases when associated with gout, arterio-sclerosis, fibroid kidney, atheroma, and age; cases of hemiplegia and paresis dependent upon vascular changes other than embolism, especially those cases which are associated in etiology with the preceding class; Bright's disease in all its forms other than acute, and albuminuria generally, and after pregnancy; rheumatoid arthritis, gout and associated lithiasis, and renal colic; cases of imperfect convalescence from acute specifics, such as scarlet fever, typhoid fever, influenza, etc., and especially if the kidneys have been involved; hepatic dyspepsia and lithæmia; and rheumatic fever after the attack. Sandwith has paid special attention to the question of hæmoptysis in Egypt, and finds that it is a rare symptom there.

It is hardly necessary to mention all the affections in which benefit may be expected from a visit to Egypt under proper supervision. But it is well to remember that there are a large number of conditions which are contra-indicated. Firstly, there are the very numerous patients in advanced consumption who are better at home, where they at least can have all the comforts that home alone can provide, and where they are surrounded by friends, and it is better to advise them to avoid the dangers and fatigues of a long journey only to land themselves in a hopeless condition amongst strangers. Any tendency to diarrhœa amongst the phthisical, convalescences from dysentery and typhoid, well-marked diabetes, and tuberculous kidneys are unsuitable conditions.

As regards heart affections, it is difficult to understand why angina pectoris, hypertrophy and dilatation of the heart aortic regurgitation and aneurysm should not benefit by residence in Egypt ; though it is necessary, of course, for these cases to be under the same careful treatment as would be considered necessary at home.

References to Literature:—

*Egypt as a Winter Resort*, F. M. Sandwith, 1889.

"Four Hundred Cases of Phthisis," F. M. Sandwith, *Lancet*, 1892, ii. 711.

*Wintering in Egypt*, A. J. M. Bentley, M.D., and C. G. Griffinhoofe, M.A., 1894.

"Wintering in Egypt," F. Peterson, M.D., *Med. Rec.*, 1892, xlii. 205.

*The Salino-Sulphurous Springs of Helouan*, Dr. Riel, 1874.

"The Influence of the Climate of Egypt upon Disease," L. Canney, M.D., *Lancet*, 1894, ii. 970.

*Helouan*, by Dr. Fenyez.

